# The bidirectional association between depression and sarcopenia: a systematic review and meta-analysis

**DOI:** 10.3389/fpubh.2025.1673755

**Published:** 2025-11-13

**Authors:** Yuan Zhu, Mengjia Pan, Yu Qiu, Zhenmei An, Shuangqing Li

**Affiliations:** 1 General Practice Medical Center, West China Hospital, Sichuan University, Chengdu, Sichuan, China; 2 Department of Endocrinology and Metabolism, West China Hospital, Sichuan University, Chengdu, Sichuan, China; 3 General Practice Medical Center, National Clinical Research Center for Geriatrics, West China Hospital, Sichuan University, Chengdu, Sichuan, China

**Keywords:** sarcopenia, depression, meta, OR, prevalence

## Abstract

**Background:**

Sarcopenia, a geriatric syndrome characterized by progressive loss of muscle mass, strength, and physical performance, elevates risks of functional decline and mortality. This study aims to investigate the co-occurrence patterns between depression and sarcopenia and characterize their epidemiological linkages.

**Methods:**

We systematically searched PubMed, Embase, Scopus, and Web of Science for studies examining depression and sarcopenia (published by July 12, 2024). Our review encompasses literature on sarcopenia prevalence, its co-occurrence with depression, and the odds ratio (OR) between these conditions. We performed statistical analyses using R.

**Results:**

Analysis of 36 studies demonstrated significantly higher pooled depression prevalence among sarcopenic individuals (0.24; 95% CI: 0.18–0.30; *I*^2^ = 95.6%) compared to non-sarcopenic controls, with sarcopenia conferring increased depression likelihood (adjusted OR = 1.49; 95% CI: 1.23–1.81; *I*^2^ = 85.8%). Similar patterns emerged for possible sarcopenia (depression prevalence: 0.15; 95% CI: 0.11–0.19; *I*^2^ = 86.4%; adjusted OR = 1.42; 95% CI: 1.10–1.83; *I*^2^ = 79.4%). Conversely, while sarcopenia prevalence was elevated in depression cohorts (0.20; 95% CI: 0.02–0.38; *I*^2^ = 94.2%), the adjusted association was non-significant (OR = 1.87; 95% CI: 0.74–4.71; *I*^2^ = 78.5%).

**Conclusion:**

Sarcopenia is significantly associated with higher depression prevalence and increased depression likelihood. While sarcopenia prevalence is elevated in depression populations, their bidirectional association requires further investigation. Future research should focus on elucidating underlying mechanisms.

**Systematic review registration:**

This review was registered with PROSPERO (number CRD42024572944).

## 1 Introduction

Sarcopenia is recognized as an age-associated disease, characterized by the diminishment of muscle mass, strength, and functionality (1). Muscle mass starts to diminish in individuals from the age of 25–30, with a notable increase in this reduction from age 50 onwards, at a pace of approximately 1% per year (2). The incidence of sarcopenia is closely linked to age, with 10%−27% of individuals older adults 60 and above being affected (3). With the increasing aging population, sarcopenia exerts significant economic pressure on healthcare systems, which is associated with a rise in health issues such as fractures, reduced mobility, diminished quality of life, and the prevalence of multiple chronic conditions (4).

Unhealthy lifestyle habits and underlying medical conditions are significant risk factors for the development of sarcopenia. An abundance of observational research has illuminated the frequent comorbidity of depression and sarcopenia in the population (5–7). Symptoms associated with depression, such as weakness, loss of appetite, reluctance, and reduced motivation, can contribute to the development of sarcopenia (8). Similarly, sarcopenia often exacerbated by factors like recurrent falls, loss of autonomy, disruptions in self-care, decreased nutrient intake, and inactivity, is linked to a heightened risk of depression. This connection results in a higher prevalence of sarcopenia among depressed individuals when compared to the general older population (9). Therefore, it implies that there is a close link between depression and sarcopenia, and clarifying the interrelationship between the two conditions would provide significant practical guidance for the prevention and treatment of these clinical diseases.

A meta-analysis conducted in 2021 indicated that the incidence of depression is high in sarcopenia, and depression is a risk factor for the development of sarcopenia (10). To further understand the relationship between depression and different states of sarcopenia, as well as whether depression and sarcopenia serve as risk factors for each other, we have updated and supplemented previous research.

## 2 Materials and methods

### 2.1 Protocol

Our view was conducted in accordance with the Preferred Reporting Items for Systematic Reviews and Meta-Analyses (PRISMA) statement (11). The protocol for the review was registered with PROSPERO (number CRD42024572944).

### 2.2 Search strategy

Two researchers independently searched the following English databases from inception to July 2024: PubMed, Embase, Scopus, and Web of Science. The following search terms were used: “sarcopenia” OR “possible sarcopenia” OR “muscle loss” OR “grip strength” OR “gait speed” AND “depression” OR “depressive symptom”. Retrieve the formula: (((((sarcopenia) OR (possible sarcopenia)) OR (muscle loss)) OR (grip strength)) OR (gait speed)) AND (depression)) OR (depressive symptom). We also screened the reference lists of all retrieved articles to identify other relevant research.

### 2.3 Eligibility criteria

The criteria for inclusion in the review are outlined below: (1) studies with a cross-sectional or cohort design; (2) studies that include populations with sarcopenia or possible sarcopenia; (3) studies with explicit diagnostic criteria for depression; and (4) studies that report on the prevalence of depression, sarcopenia, and odds ratios (ORs). The exclusion criteria are as follows: (1) studies from which data cannot be extracted; (2) articles not published in English; and (3) case reports, editorial letters, abstracts, and review articles.

### 2.4 Study selection

Two researchers conducted an independent literature selection process. Initially, duplicates were eliminated using Endnote X9 software. Subsequently, the titles and abstracts of the remaining articles were reviewed. The full texts were then examined to determine the eligibility of each study for inclusion. The rationale for excluding articles during the second and final stages was documented. In cases of disagreement, a third researcher was consulted to resolve the discrepancies.

### 2.5 Sarcopenia and depression diagnosis criteria

Sarcopenia was defined according to the Asian Working Group for Sarcopenia (AWGS 2014/2019), the European Working Group on Sarcopenia in Older People (EWGSOP 2010), or the Finnish Health 2000 (FINH) criteria; depression was assessed using the Self-Rating Depression Scale (SDS), 15-item Geriatric Depression Scale (GDS-15), 10-item Center for Epidemiologic Studies Depression Scale (CES-D-10), Brief Child and Family Interview for Clinical Referral (B-CIS-R), or Diagnostic and Statistical Manual of Mental Disorders (DSM)-based tools.

### 2.6 Data extraction

A systematic approach was employed to extract data from the studies, encompassing details such as the lead author, year of publication, country, study design, age, number of patients with sarcopenia or possible sarcopenia, number of patients with depression among those with sarcopenia, events prevalence, and the methods used to diagnosis sarcopenia and depression. The primary outcomes of interest were the prevalence of depression in individuals with sarcopenia or possible sarcopenia and the crude and adjusted associations between depression and sarcopenia or possible sarcopenia, quantified using odds ratios (ORs) and 95% confidence intervals (CIs). Additionally, another key outcome was the prevalence of sarcopenia in those with depression and the crude and adjusted associations between sarcopenia and depression. When several adjusted logistic regression models were reported, we extracted the one most commonly presented.

### 2.7 Quality assessment under such circumstances, we employed a fixed-effects model under such circumstances, we employed a fixed-effects model

The two researchers independently appraised the quality of each study using the Newcastle–Ottawa Scale, a well-established tool for assessing the quality of cross-sectional studies. For cohort or case-control studies, the maximum achievable score was 9, while for cross-sectional studies, it was 6, with higher scores reflecting superior methodological quality (12). Any discrepancies in scoring between the researchers were addressed through collaborative discussion (Supplementary Table S3).

### 2.8 Statistical analysis

Data analysis was conducted utilizing R version 4.4.1. The degree of heterogeneity among studies was assessed through the *I*^2^ statistic, a metric that quantifies the degree of variation in study results. Typically, an *I*^2^ value ranging from 0 to 50% indicates minimal statistical heterogeneity. We employed a fixed-effects model in R to amalgamate data on continuous variables. In contrast, studies exhibiting an *I*^2^ value exceeding 50% were classified as having substantial heterogeneity, necessitating the application of a random-effects model coupled with a heterogeneity test within the R.

### 2.9 Subgroup analysis

We explored heterogeneity by conducting subgroup analyses based on depression and sarcopenia diagnostic criteria, BMI, and country. BMI thresholds for overweight and obesity were set at ≥24.0 and ≥28.0, respectively.

### 2.10 Sensitivity analysis

With an adequate number of studies identified, we planned to assess result robustness via sensitivity analysis, omitting research focused on special populations.

## 3 Results

### 3.1 Search results

**Figure 1:**
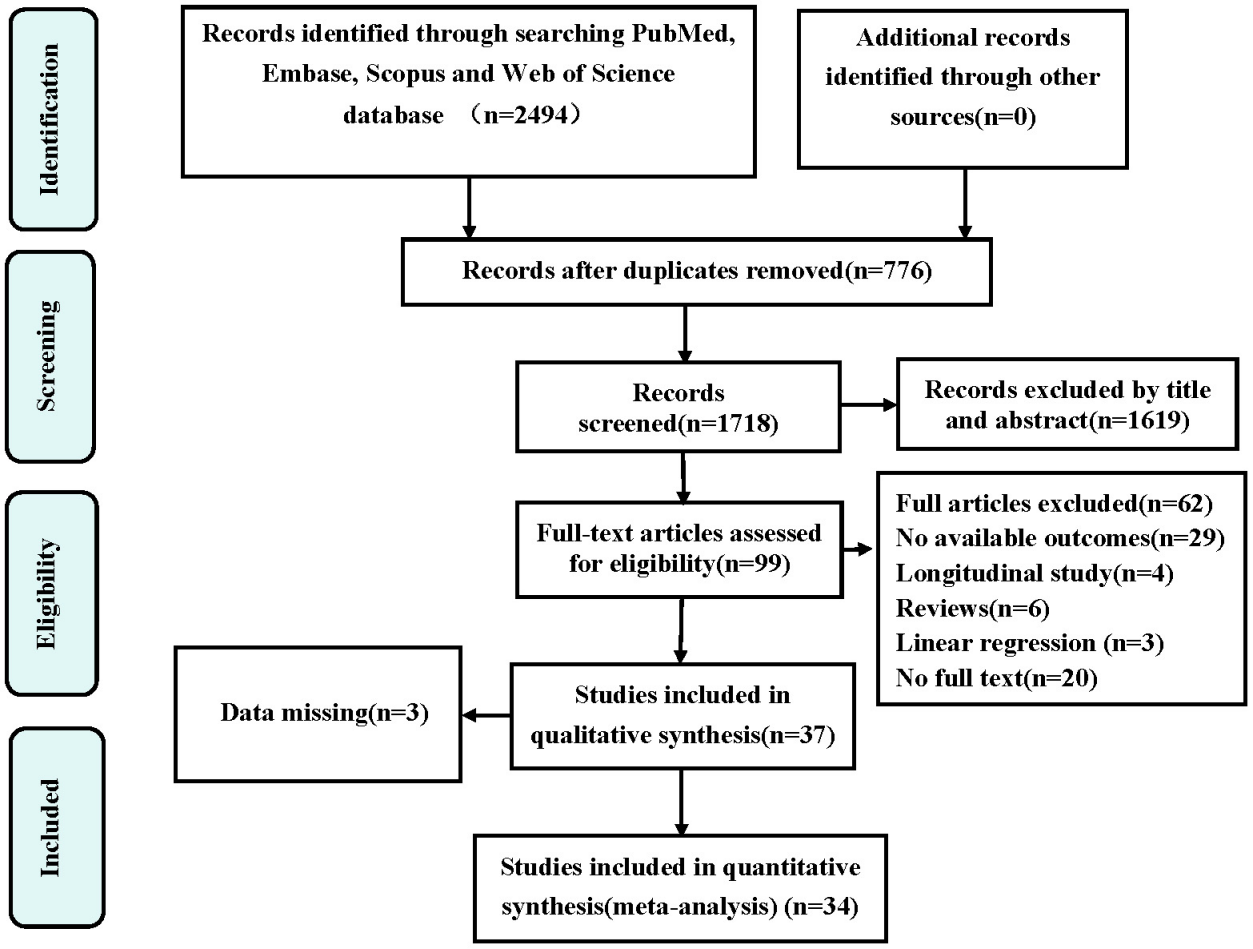
Preferred reporting items for systematic reviews and meta-analyses (PRISMA) flow diagram for the study selection. OR, odds ratio.

Our initial literature search yielded 2,494 articles, leaving 776 after removing duplicates. Screening of titles and abstracts led to the selection of 99 studies for full-text review. Subsequently, 62 articles were excluded and three omitted for missing data, leaving us with 34 publications (8, 13–45) that met our criteria. Among these, three (13, 14, 36) focused on depression prevalence in sarcopenia only, seven (15–21) reported ORs between depression and sarcopenia only, and fourteen (8, 22–34) investigated both depression prevalence in sarcopenia and ORs between depression and sarcopenia. Moreover, one (37) study involved depression prevalence in possible sarcopenia only, and six (29, 35, 38–41) explored both depression prevalence in possible sarcopenia and its ORs. Additionally, one (42) study involved sarcopenia prevalence in depression only, one (43) reported ORs between sarcopenia and depression only, and two (44, 45) investigated both sarcopenia prevalence in depression and ORs between sarcopenia and depression. The detailed selection process is presented in Figure 1.

### 3.2 Prevalence and OR of depression in sarcopenia

#### 3.2.1 Study characteristics between depression and sarcopenia

Table 1 synthesizes the characteristics of 17 studies assessing depression prevalence in sarcopenia. The mean age of participants across studies varied from 54.7 to 88.6 years. The majority of the studies included in our analysis were conducted in China and Japan, all of which used a cross-sectional design. Furthermore, three of these studies conducted stratified analyses by sex.

**Table 1:** Characteristics of studies included in the meta-analysis for prevalence of depression in sarcopenia.

**First author**	**Country**	**Study design**	**Age (mean)**	**No. of sarcopenia**	**No. of depression**	**Prevalence**	**BMI (mean)**	**Sarcopenia diagnosis**	**Depression diagnosis**
Byeon 2016	Korean	Cross-section	54.7	319	17	0.05	27.9	AWGS (2014)	SDS
Chen 2021	China	Cross-section	72.5	80	9	0.11	22.1	AWGS (2019)	GDS-15
Chen 2021	China	Cross-section	71.9	92	8	0.09	22.4	AWGS (2019)	GDS-15
Darroch 2021	Newland	Cross-section	88.6	37	14	0.38	21.6	EWGSOP (2010)	GDS-15
Endo 2021	Japanese	Cross-section	80.0	30	12	0.40	20.1	AWGS (2014)	SDS
Gao 2021	China	Cross-section	72.0	769	276	0.36	Unknown	AWGS (2019)	CES-D-10
Hayashi 2019	Japanese	Cross-section	74.0	41	18	0.44	21.0	AWGS (2014)	GDS-15
Li 2024	China	Cross-section	72.0	633	203	0.32	Unknown	AWGS (2019)	CES-D-10
Lu 2023	China	Cross-section	73.2	132	11	0.08	21.3	AWGS (2019)	GDS-15
Kitamura 2021	Japanese	Cross-section	78.0	105	31	0.30	21.4	AWGS (2019)	GDS-15
Kitamura 2021	Japanese	Cross-section	78.0	156	66	0.43	21.4	AWGS (2019)	GDS-15
Kilavuz 2018	Turkey	Cross-section	72.0	40	13	0.33	Unknown	EWGSOP (2010)	GDS-15
Szlejf 2019	Brazil	Cross-section	61.0	114	10	0.08	27.0	FNIH	B-CIS-R
Sugimoto 2016	Japanese	Cross-section	80.0	88	36	0.41	19.6	AWGS (2014)	GDS-15
Ishii 2016	Japanese	Cross-section	77.0	64	17	0.27	Unknown	EWGSOP (2010)	GDS-15
Ishii 2016	Japanese	Cross-section	77.0	236	26	0.11	Unknown	EWGSOP (2010)	GDS-15
Huang 2015	China	Cross-section	77.0	50	4	0.08	24.7	AWGS (2014)	CES-D-10
Alexandre 2014	Brazil	Cross-section	70.0	266	36	0.14	21.0	EWGSOP (2010)	GDS-15
Hsu 2014	China	Cross-section	84.0	109	32	0.30	20.9	EWGSOP (2010)	GDS-15
Landi 2012	Italy	Cross-section	87.0	66	20	0.30	23.8	EWGSOP (2010)	DSM

AWGS, Asian Working Group for Sarcopenia; EWGSOP, European Working Group on Sarcopenia in Older People; FNIH, Foundation for the National Institutes of Health; SDS, Self-rating Depression Scale; GDS, Geriatric Depression Scale; CES-D, Center for Epidemiologic Studies Depression Scale; B-CIS-R, Brazilian version of the Clinical Interview Scheduled Revised; DSM, Diagnostic and Statistical Manual of Mental Disorders.

Table 2 presents a comprehensive overview of the 20 studies that reported on the odds ratios (ORs) associated with depression in the context of sarcopenia, encompassing a total of 34,029 participants. The age demographic of these studies spanned a broad spectrum, with mean ages of the participants ranging from 38.2 to 86.0 years, indicating a diverse representation across adulthood. Furthermore, three of these studies conducted stratified analyses by sex.

**Table 2:** Characteristics of studies included in the meta-analysis for ORs between depression and sarcopenia.

**First author**	**Country**	**Study design**	**Age (mean)**	**Sample size**	**BMI (mean)**	**OR (95%CI)**	**Adjusted OR (95%CI)**	**Sarcopenia diagnosis**	**Depression diagnosis**
Byeon 2016	Korean	Cross-section	49.5	7,364	25.7	1.54 (0.87–2.73)	1.12 (0.59–2.13)	EWGSOP (2010)	SDS
Darroch 2021	Newland	Cross-section	86.0	91	24.9	0.90 (0.70, 1.00)	0.80 (0.60, 1.10)	EWGSOP (2010)	GDS-15
Endo 2021	Japanese	Cross-section	80.0	155	22.8	/	1.05 (0.99–1.11)	AWGS (2014)	SDS
Gao 2021	China	Cross-section	72.0	769	Unknown	/	1.64 (1.23–2.19)	AWGS (2019)	CES-D-10
Hayashi 2019	Japanese	Cross-section	72.5	432	22.9	/	2.38 (1.18–4.18)	AWGS (2014)	GDS-15
Li 2024	China	Cross-section	72.0	633	Unknown	1.39 (1.18, 1.64)	1.30 (1.03, 1.63)	AWGS (2019)	CES-D-10
Lu 2023	China	Cross-section	72.6	452	24.7	2.30 (0.87, 6.09)	1.63 (0.52, 5.08)	AWGS (2019)	GDS-15
Lu 2023	China	Cross-section	71.2	667	24.6	0.67 (1.26.1.74)	0.49 (0.17, 1.40)	AWGS (2019)	GDS-15
Zhong 2023	China	Cross-section	65.7	2,851	Unknown	1.49 (1.37, 1.62)	1.24 (1.08, 1.42)	AWGS (2019)	CES-D-10
Kitamura 2021	Japanese	Cross-section	78.0	917	23.4	/	1.20 (0.70, 2.10)	AWGS (2019)	GDS-15
Kilavuz 2018	Turkey	Cross-section	72.2	861	Unknown	/	2.55 (1.11, 1.58)	EWGSOP (2010)	GDS-15
Szlejf 2019	Brazil	Cross-section	61.0	5,927	27.0	2.30 (1.19, 4.46)	2.23 (1.11, 4.48)	FNIH	B-CIS-R
Sugimoto 2016	Japanese	Cross-section	77.0	139	21.8	/	2.11 (0.90, 4.93)	AWGS (2014)	GDS-15
Sugimoto 2016	Japanese	Cross-section	77.0	279	21.8	/	1.24 (0.66, 1.32)	AWGS (2014)	GDS-15
Ishii 2016	Japanese	Cross-section	77.0	1,732	Unknown	/	2.79 (1.43, 5.43)	EWGSOP (2010)	GDS-15
Ishii 2016	Japanese	Cross-section	77.0	1,732	Unknown	/	0.93 (0.55, 1.60)	EWGSOP (2010)	GDS-15
Hsu 2014	China	Cross-section	82.7	353	23.0	2.55 (1.41, 4.60)	2.25 (1.03, 4.89)	EWGSOP (2010)	GDS-15
Kim 2014	Korean	Cross-section	63.9	95	22.3	8.75 (2.74, 27.9)	6.87 (2.06, 22.96)	EWGSOP (2010)	BDI-II
Yuenyong 2021	Thailand	Cross-section	60.0	104	23.5	/	3.33 (1.14, 9.16)	AWGS (2019)	BDI-II
Yuenyong 2020	Thailand	Cross-section	67.0	330	25.6	2.34 (1.22, 4.45)	2.09 (1.06, 4.13)	AWGS (2014)	GDS-15
Fábrega 2020	Spain	Cross-section	72.0	304	29.1	/	1.10 (1.02, 1.19)	AWGS (2019)	HADS
Patino 2017	USA	Cross-section	76.0	1,509	Unknown	1.25 (0.81, 1.94)	0.82 (0.50, 1.35)	EWGSOP (2010)	GDS-15
Nan 2023	USA	Cross-section	38.2	6,603	Unknown	2.86 (1.68, 4.85)	2.97 (1.73, 5.11)	FNIH	PHQ9

AWGS, Asian Working Group for Sarcopenia; EWGSOP, European Working Group on Sarcopenia in Older People; FNIH, Foundation for the National Institutes of Health; SDS, Self-rating Depression Scale; GDS, Geriatric Depression Scale; CES-D, Center for Epidemiologic Studies Depression Scale; B-CIS-R, Brazilian version of the Clinical Interview Scheduled Revised; BDI-II, Beck Depression Inventory II; HADS, Hospital Anxiety and Depression Scale; PHQ9, Patient Health Questionnaire-9.

#### 3.2.2 Meta-analysis results

**Figure 2:**
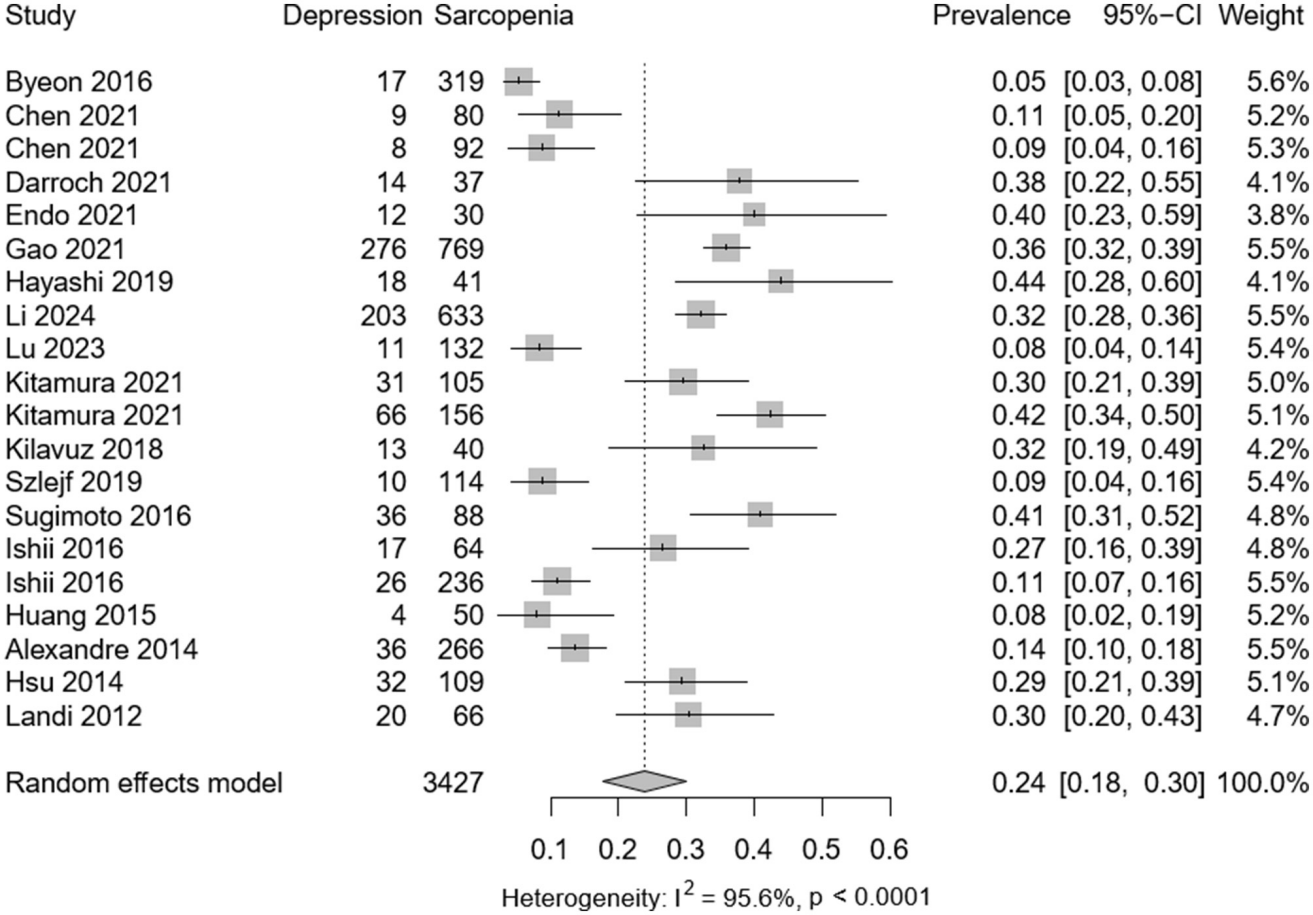
Forest plot of depression in sarcopenia. CI, confidence interval; OR, odds ratio.

**Figure 3:**
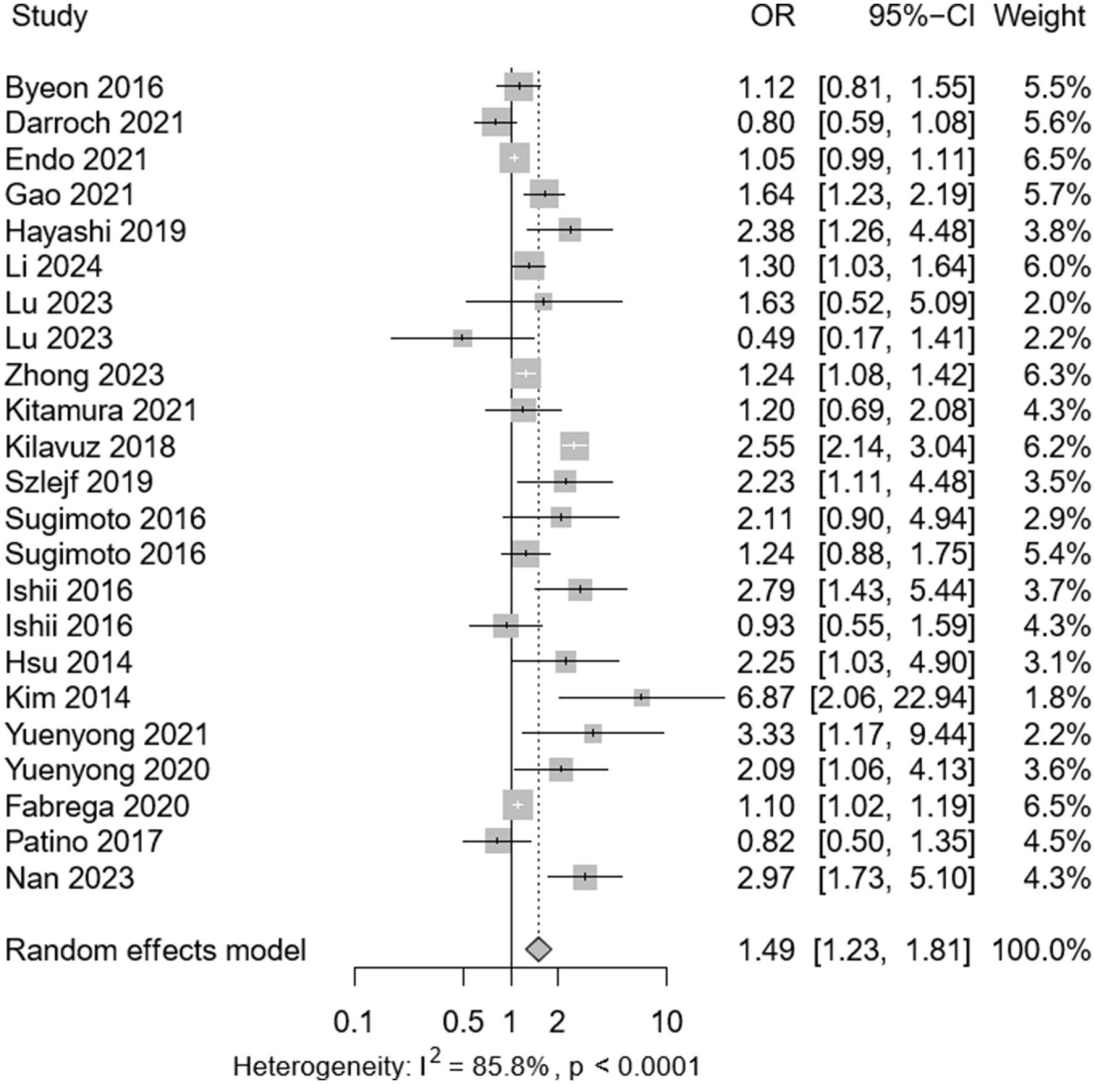
Forest plot of the adjusted odds ratio (ORs) between depression and sarcopenia. CI, confidence interval; OR, odds ratio.

Among the selected studies, 3,427 individuals presented with sarcopenia and 859 with depression. Meta-analysis revealed a pooled prevalence of depression in sarcopenic patients at 0.24 (95% CI: 0.08–0.30), with significant heterogeneity observed (*P* < 0.001; *I*^2^ = 95.6%) as depicted in Figure 2. Among 34,029 participants (mean age 62.1 years), the adjusted OR for the association between depression and sarcopenia was 1.49 (95% CI: 1.23–1.81), as shown in Figure 3. Moderate heterogeneity was noted in the adjusted ORs across studies (*P* < 0.001; *I*^2^ = 85.8%).

#### 3.2.3 Subgroup analyses

Subgroup analysis indicated a range of depression prevalence among sarcopenia patients, as determined by various diagnostic criteria range from 0.22 (95% CI: 0.01–0.55) by SDS to 0.27 (95% CI: 0.09–0.44) by AWGS 2014. Furthermore, the prevalence was 0.19 (95% CI: 0.10–0.29) in China and 0.27 (95% CI: 0.15–0.39) in Japanese (Supplementary Table S4a).

Subgroup analysis results indicated an adjusted OR of 1.50 (95% CI: 1.05–2.13) for depression associated with sarcopenia diagnosed by AWGS 2014 criteria, and an OR of 1.25 (95% CI: 1.10–1.14) by AWGS 2019 criteria (Supplementary Table S4b).

#### 3.2.4 Sensitivity analysis

Sugimoto et al. (24) studied patients with amnestic mild cognitive impairment or Alzheimer's disease, whereas Hsu et al.'s (30) research involved male veterans from the same community. After excluding these studies in a sensitivity analysis, the pooled prevalence showed minimal change, confirming the robustness of our meta-analysis (Supplementary Figure S3a). Likewise, we excluded four studies that contributed ORs: Sugimoto et al. (24) work with patients having amnestic mild cognitive impairment or Alzheimer's, Hsu et al.'s (30) research involving veterans, and Yuenyong et al.' (15) and Kim et al.'s (17) examination of end-stage renal disease patients on hemodialysis. The sensitivity analysis indicated that the pooled adjusted ORs remained stable (Supplementary Figure S3b).

### 3.3 Prevalence and OR of depression in possible sarcopenia

#### 3.3.1 Study characteristics between depression and possible sarcopenia

Supplementary Table S1 consolidates data from seven studies evaluating depression rates in individuals with possible sarcopenia, with participant ages ranging from 40.0 to 78.0 years. Predominantly Chinese and Korean, these studies uniformly adopted a cross-sectional methodology. Notably, one included sex-based stratification, differentiating between menopausal and non-menopausal groups.

Supplementary Table S2 provides a succinct summary of seven studies examining the odds ratios (ORs) linking depression with possible sarcopenia, involving 7,450 participants. Notably, one of these studies included stratified analyses by sex and menopausal status.

#### 3.3.2 Meta-analysis results

Among the selected studies, 2,992 individuals presented with possible sarcopenia and 405 with depression. Meta-analysis revealed a pooled prevalence of depression in possible sarcopenia patients at 0.15 (95% CI: 0.11–0.19), with significant heterogeneity observed (*P* < 0.001; *I*^2^ = 86.4%) as depicted in Supplementary Figure S1. Among 7,450 participants (mean age 64.3 years), the adjusted OR for the association between depression and sarcopenia was 1.42 (95% CI: 1.10–1.83), as shown in Supplementary Figure S2. Moderate heterogeneity was noted in the adjusted ORs across studies (*P* < 0.001; *I*^2^ = 79.4%).

#### 3.3.3 Subgroup analyses

Subgroup analysis of possible sarcopenia patients revealed depression prevalence rates of 0.15 (95% CI: 0.07–0.22) under AWGS 2014, 0.14 (95% CI: 0.05–0.24) under AWGS 2019, and 0.12 (95% CI: 0.04–0.19) using PHQ-9. China reported a higher prevalence at 0.17 (95% CI: 0.10–0.24), while Japan had a rate of 0.10 (95% CI: 0.08–0.23), as detailed in Supplementary Table S4c. However, the ORs analysis for depression associated with possible sarcopenia showed no significant differences, as noted in Supplementary Table S4d.

#### 3.3.4 Sensitivity analysis

Heo et al. (38) included both premenopausal and postmenopausal women with varying estrogen levels. After exclusion, pooled incidence and ORs remained virtually unchanged (Supplementary Figures S3c, d), confirming robustness.

### 3.4 Prevalence and OR of sarcopenia in depression

#### 3.4.1 Study characteristics between sarcopenia and depression

Supplementary Table S5a summarizes findings from three cross-sectional studies on sarcopenia prevalence among depressed individuals, with ages spanning 67.7–75.8 years, primarily from Turkey, including a sex-stratified analysis in one study. Supplementary Table S5b offers a concise overview of three studies on the ORs connecting sarcopenia to depression, encompassing 1,370 participants older adults 68.7–74.3 years.

#### 3.4.2 Meta-analysis results

Among the selected studies, 256 individuals presented with depression and 54 with sarcopenia. Meta-analysis revealed a pooled prevalence of sarcopenia in depression patients at 0.20 (95% CI: 0.02–0.38), with significant heterogeneity observed (*P* < 0.001; *I*^2^ = 94.2%) as depicted in Supplementary Figure S4a. Encompassing 1,370 subjects with an average age of 70.7 years, the adjusted OR for the association between depression and sarcopenia was 1.87 (95% CI: 0.74–4.71), as shown in Supplementary Figure S4b. Moderate heterogeneity was noted in the adjusted ORs across studies (*P* < 0.001; *I*^2^ = 78.5%).

## 4 Discussion

The present study aimed to compile the latest evidence regarding the prevalence of depression in patients with sarcopenia or possible sarcopenia, as well as the prevalence of sarcopenia among those with depression, and to assess the correlation between sarcopenia and depression. The results revealed a high prevalence of depression among sarcopenia patients and a positive correlation between depression and sarcopenia or possible sarcopenia, which persisted after adjusting for relevant covariates.

Our research findings are consistent with previous meta-analyses on the correlation between sarcopenia and depression (10), confirming a significant positive correlation between the two. This study also found that the incidence of depression is higher among individuals with possible sarcopenia, and the positive correlation between depression and sarcopenia remains significant after controlling for confounding factors. However, despite the high prevalence of sarcopenia in patients with depression, our results suggest that sarcopenia may not be a risk factor for depression. This conclusion may be influenced by the limited literature in this field, which could interfere with the interpretation of the research findings.

In the subgroup meta-analysis stratified by country, diagnostic criteria for sarcopenia or possible sarcopenia, significant disparities were observed between subgroups. The utilization of AWGS 2014 criteria resulted in a higher prevalence of depression in sarcopenic patients than the AWGS 2019 criteria, and the correlation between depression and sarcopenia was more pronounced with the earlier criteria. The divergent diagnostic thresholds and assessment methodologies between AWGS 2014 and AWGS 2019 may account for the varying assessments of the sarcopenia-depression association (46, 47). The lower diagnostic thresholds of the AWGS 2014 criteria may enhance sensitivity, facilitating the detection of associations between sarcopenia and depression by encompassing a broader range of potential cases (47, 48). Nevertheless, this could concurrently diminish specificity, increasing the risk of false positives and potentially diluting the genuine strength of the sarcopenia-depression association. Namely, the findings of this study suggest that the updated criteria in AWGS 2019 may enhance the precision of sarcopenia diagnosis and the credibility of assessing the relationship between depression and sarcopenia. Hence, the selection of appropriate diagnostic criteria is essential for research on sarcopenia and its association with depression. In conclusion, the selection of diagnostic criteria is pivotal for investigations into sarcopenia and its correlation with depression.

The use of different depression scales significantly impacts the assessment of depression prevalence in sarcopenic or possibly sarcopenic patients, with notable differences between subgroups. Most studies employed the GDS-15 and CES-D-10 scales, with less frequent use of others. The CES-D-10 yielded a slightly higher prevalence of depression than the GDS-15. Meta-analysis revealed that significant correlations between depression and sarcopenia persisted in subgroups analyzed with the GDS-15, but not with the CES-D-10. This confirms that tool choice materially influences prevalence estimates and suggests GDS-15 is superior to CES-D-10 for detecting depression in this setting (49).

Despite subgroup and sensitivity analyses, considerable heterogeneity remained in the meta-analysis, likely due to several factors: first, although sarcopenia was diagnosed in the included studies, there was inconsistency in diagnostic criteria and no grading of sarcopenia severity, leading to potential variability in sarcopenia severity across studies (50). Second, most studies utilized GDS-15 or CES-D-10 questionnaires for depressive symptom assessment, which are screening tools rather than diagnostic instruments, and may associate depressive symptoms with physical illnesses (51). Additionally, the severity of depression among study participants was not uniform, inevitably introducing self-report and recall biases that could affect the prevalence of sarcopenia and depressive symptoms to varying extents (52). Third, while ORs were adjusted for demographic data including age, gender, BMI, and education, numerous confounders such as disability, frailty, physical activity, and sex hormones might have influenced the association between sarcopenia and depression, and these factors could not be fully harmonized in this study. Finally, differences in ethnicity, region, dietary habits, medical conditions, and quality control in the research process between studies may contribute to greater heterogeneity (53). Therefore, future studies should, whenever feasible, adopt gold-standard diagnostic instruments for depression (e.g., DSM or PHQ-9) to minimize false-positive rates and ensure diagnostic validity.

Substantial heterogeneity exists in sarcopenia diagnostic criteria (primarily AWGS 2014/2019 vs. EWGSOP 2010), yet our study demonstrates robust depression prevalence (0.24–0.27) and consistent depression-sarcopenia association (OR = 1.25–1.66). Nevertheless, these variations pose dual challenges for clinical translation and research synthesis: quantitative analyses indicate that overly inclusive criteria (e.g., EWGSOP 2010) increase false-positive risks, potentially leading to unnecessary antidepressant treatment in mild cases, whereas excessively stringent definitions (e.g., AWGS 2019 requiring concurrent low muscle strength, poor physical function, and reduced muscle mass) may miss high-risk individuals with isolated strength impairment. To optimize clinical pathways, we propose a stepped diagnostic framework: primary care settings should prioritize EWGSOP criteria with GDS-15 scale (high-sensitivity screening), while specialists (e.g., psychiatrists and endocrinologists) in tertiary institutions should apply AWGS 2019 criteria with PHQ-9 instrument (high-specificity intervention).

Our study found a significant positive correlation between depression and sarcopenia, which persisted after controlling for confounding factors. Therefore, elucidating the causal relationship between depression and sarcopenia is of great importance. Although two studies have further confirmed the positive correlation between depression and sarcopenia using Mendelian randomization methods (21, 54). One study showed a negative correlation between depression and sarcopenia (55). Additionally, two studies indicated no significant association between sarcopenia and major depressive disorder (6, 56). These conflicting findings suggest that their relationship is modulated by multidimensional factors, among which the mediating role of dietary patterns is particularly crucial. Emerging evidence indicates that nutritional profiles, particularly protein and micronutrient levels, may directly influence depression-related muscle metabolic dysregulation and indirectly affect this relationship through pathways involving inflammatory status and gut microbiota (57). This modulation exhibits population heterogeneity: diets rich in antioxidant nutrients may buffer depression-induced muscle catabolism, whereas malnutrition coupled with depression may accelerate sarcopenic progression. In summary, future research should integrate multi-omics technologies to systematically elucidate the mechanistic roles of mediating variables, including nutrition, physical activity, and comorbidities, while standardizing methodological approaches.

In the current meta-analysis, we are confident that our literature search was comprehensive, with no relevant studies missed. We conducted study selection, data extraction, and quality assessment to minimize errors. We supplemented and updated the literature from previous meta-analyses. Despite encouraging results, the study has several limitations. Firstly, all selected articles were cross-sectional, preventing the establishment of a causal relationship between sarcopenia and depression. Secondly, substantial heterogeneity was observed across the included studies regarding diagnostic methodologies for sarcopenia and depression, geographic distribution, and BMI parameters. Despite systematic exploration through subgroup analyses, residual heterogeneity remained elevated (*I*^2^ > 80%), suggesting that unmeasured confounders may compromise the robustness of the findings. Consequently, caution should be exercised when extrapolating these results to broader clinical contexts. Thirdly, no subgroup analysis was conducted based on the severity of sarcopenia and depression.

## 5 Conclusion

Sarcopenia is significantly associated with higher depression prevalence and increased depression likelihood. While sarcopenia prevalence is elevated in depression populations, their bidirectional association requires further investigation. Future research should focus on elucidating underlying mechanisms.

## Data Availability

The original contributions presented in the study are included in the article/Supplementary material, further inquiries can be directed to the corresponding authors.
