# Supplementary material for: The bidirectional association between depression and sarcopenia: a systematic review and meta-analysis
Source: Front Public Health. 2025 Nov 13;13:1673755. doi: 10.3389/fpubh.2025.1673755 (PMC12658358; doi:10.3389/fpubh.2025.1673755)
Supplement: Supplementary file 1 [file Table_1.docx]

Table S1 Characteristics of studies included in the meta-analysis for prevalence of depression in possible sarcopenia

| First author | Country | Study design | Age  (mean) | No. of Possible sarcopenia | No. of  Depression | Prevalence | BMI  (mean) | Possible  sarcopenia diagnosis | Depression  diagnosis |
| --- | --- | --- | --- | --- | --- | --- | --- | --- | --- |
| Heo 2018  Heo 2018  Heo 2018  Lee 2022  Lu 2023  Tian 2022  Vesconcelos 2016  Li 2024  Endo 2021 | Korean Korean  Korean  Korean  China  China  Brazil  China  Japanese | Cross-section  Cross-section  Cross-section  Cross-section  Cross-section  Cross-section  Cross-section  Cross-section  Cross-section | 52.2  40.0  57.1  74.1  73.8  69.0  74.2  72.4  78.0 | 383  265  460  538  213  195  315  498  125 | 35  31  59  42  25  48  53  78  34 | 0.09  0.12  0.13  0.08  0.12  0.25  0.17  0.16  0.27 | 22.7  20.6  21.7  Unknown  25.7  Unknown  Unknown  Unknown  19.9 | AWGS (2014)  AWGS (2014)  AWGS (2014)  AWGS (2019)  AWGS (2019)  AWGS (2019)  EWGSOP (2010)  EWGSOP (2010)  AWGS (2014) | BDI-II  BDI-II  BDI-II  PHQ-9  GDS-15  CES-D-10  GDS-15  PHQ-9  SDS |

Abbreviations: AWGS, Asian Working Group for Sarcopenia; EWGSOP, European Working Group on Sarcopenia in Older People; SDS, Self-rating Depression Scale; GDS, Geriatric Depression Scale; CES-D, Center for Epidemiologic Studies Depression Scale; BDI-II, Beck Depression Inventory II; HADS, Hospital Anxiety and Depression Scale; PHQ9, Patient Health Questionnaire-9.
