# Supplementary material for: The bidirectional association between depression and sarcopenia: a systematic review and meta-analysis
Source: Front Public Health. 2025 Nov 13;13:1673755. doi: 10.3389/fpubh.2025.1673755 (PMC12658358; doi:10.3389/fpubh.2025.1673755)
Supplement: Supplementary file 2 [file Table_2.docx]

Table S2 Characteristics of studies included in the meta-analysis for ORs between depression and possible sarcopenia

| First author | Country | Study design | Age  (mean) | Sample  size | BMI  (mean) | OR (95%CI) | Adjust OR (95%CI) | Possible sarcopenia diagnosis | Depression diagnosis |
| --- | --- | --- | --- | --- | --- | --- | --- | --- | --- |
| Chen 2022  Heo 2018  Heo 2018  Heo 2018  Lee 2022  Lu 2023  Lu 2023  Vesconcelos 2016  Li 2024  Endo 2021 | China  Korean  Korean  Korean  Korean  China  China  Brazil  China  Japanese | Cross-section  Cross-section  Cross-section  Cross-section  Cross-section  Cross-section  Cross-section  Cross-section  Cross-section  Cross-section | 70.0  52.2  40.0  57.1  74.1  72.6  71.2  74.2  72.8  78.0 | 1860  383  265  460  538  452  667  1374  698  753 | 22.6  22.7  20.6  21.7  Unknown  24.7  24.6  Unknown  Unknown  19.9 | /  3.56(1.63,7.77)  0.86(1.47,1.56)  1.34(0.85,2.13)  /  1.08(0.36,3.27)  1.87(0.04,3.24)  /  1.53(0.96,2.44)  / | 1.69(1.32,2.17)  3.91(1.63,9.39)  0.71(0.32,1.57)  1.64(0.87,2.52)  2.06(1.36,3.11)  0.68(0.19,2.44)  1.84(1.02,3.34)  0.99(0.71,1.39)  1.56(0.85,2.84)  1.03(1.00,1.06) | AWGS (2019)  AWGS (2014)  AWGS (2014)  AWGS (2014)  AWGS (2019)  AWGS (2019)  AWGS (2019)  EWGSOP (2010)  EWGSOP (2010)  AWGS (2014) | SDS  BDI-II  BDI-II  BDI-II  PHQ-9  GDS-15  GDS-15  GDS-15  PHQ-9  SDS |

Abbreviations: AWGS, Asian Working Group for Sarcopenia; EWGSOP, European Working Group on Sarcopenia in Older People; SDS, Self-rating Depression Scale; GDS, Geriatric Depression Scale; BDI-II, Beck Depression Inventory II; PHQ9, Patient Health Questionnaire-9.
