# Supplementary material for: The bidirectional association between depression and sarcopenia: a systematic review and meta-analysis
Source: Front Public Health. 2025 Nov 13;13:1673755. doi: 10.3389/fpubh.2025.1673755 (PMC12658358; doi:10.3389/fpubh.2025.1673755)
Supplement: Supplementary file 3 [file Table_3.docx]

Table S3: Risk of bias of the included studies using the Newcastle-Ottawa Scale.

| First author | Selection | | | | Comparability | Outcome | | |
| --- | --- | --- | --- | --- | --- | --- | --- | --- |
| Byeon 2016  Chen 2021  Darroch 2021  Endo 2021  Gao 2021  Hayashi 2019  Li 2024  Lu 2023  Kitamura 2021  Kilavuz 2018  Szlejf 2019  Sugimoto 2016  Ishii 2016  Huang 2015  Alexandre 2014  Hsu 2014  Landi 2012  Zhong 2023  Kim 2014  Yuenyong 2021  Yuenyong 2020  Fábrega 2020  Patino 2017  Nan 2023  Heo 2018  Lee 2022  Tian 2022  Vesconcelo2016  Li 2024  Chen 2022  Yazar 2019  Wang 2018  Delibas 2021  Lee 2018 | Q1  *  *  *  *  *  *  *  *  *  *  *  *  *  *  *  *  *  *  *  *  *  *  *  *  *  *  *  *  *  *  *  *  *  * | Q2  *  *  *  *  *  *  *  *  *  *  *  *  *  *  *  *  *  *  *  *  *  *  *  *  *  *  *  *  *  *  *  *  *  * | Q3  *  *  *  *  *  *  *  *  *  *  *  *  *  *  *  *  *  *  *  *  *  *  *  *  *  *  *  *  *  *  *  * | Q4  *  *  *  *  *  *  *  *  *  *  *  *  *  *  *  *  *  *  *  *  *  *  *  *  *  *  *  *  *  *  *  *  *  * | Q1  **  *  **  **  **  **  **  **  **  **  **  **  **  *  *  **  *  **  **  **  **  **  **  **  *  **  **  *  **  **  *  *  *  ** | Q1  **  **  **  **  **  **  **  **  **  **  **  **  **  **  **  **  **  **  **  **  **  **  **  **  **  **  **  **  **  **  **  **  ** | Q2  *  *  *  *  *  *  *  *  *  *  *  *  *  *  *  *  *  *  *  *  *  *  *  *  *  *  *  *  *  *  *  *  * | Q3 |

NEWCASTLE - OTTAWA QUALITY ASSESSMENT SCALE

CASE CONTROL STUDIES

Note: A study can be awarded a maximum of one star for each numbered item within the Selection and Exposure categories. A maximum of two stars can be given for Comparability.

**Selection**

Q1:Is the case definition adequate?

a) yes, with independent validation

b) yes, eg record linkage or based on self reports

c) no description

Q2:Representativeness of the cases

a) consecutive or obviously representative series of cases

b) potential for selection biases or not stated

Q3: Selection of Controls

a) community controls

b) hospital controls

c) no description

Q4:Definition of Controls

a) no history of disease (endpoint)

b) no description of source

**Comparability**

Q1:Comparability of cases and controls on the basis of the design or analysis

a) study controls for ______________ (Select the most important factor.)

b) study controls for any additional factor. (This criteria could be modified to indicate specific control for a second important factor.)

**Exposure**

Q1:Ascertainment of exposure

a) secure record (eg surgical records)

b) structured interview where blind to case/control status

c) interview not blinded to case/control status

d) written self report or medical record only

e) no description

Q2: Same method of ascertainment for cases and controls

a) yes

b) no

Q3: Non-Response rate

a) same rate for both groups

b) non respondents described

c) rate different and no designation
