# Supplementary material for: The bidirectional association between depression and sarcopenia: a systematic review and meta-analysis
Source: Front Public Health. 2025 Nov 13;13:1673755. doi: 10.3389/fpubh.2025.1673755 (PMC12658358; doi:10.3389/fpubh.2025.1673755)
Supplement: Supplementary file 4 [file Table_4.docx]

Table S4a The results of subgroup analysis in prevalence of depression in sarcopenia

| Outcomes | No. of studies | Meta-analysis results | *I*^2^ | *P* |
| --- | --- | --- | --- | --- |
| Diagnostic criteria for sarcopenia  AWGS (2014)  AWGS (2019)  EWGSOP (2010)  FNIH  Measurement method for depression  SDS  GDS-15  CES-D-10  B-CIS-R  DSM  Body Mass Index  Normal  Overweight  Unknown  Country  China  Korean  Newland  Japanese  Turkey  Brazil  Italy | 5  5  6  1  2  10  3  1  1  10  3  4  6  1  1  5  1  2  1 | 0.27(0.09-0.44)  0.24(0.13-0.35)  0.24(0.16-0.32)  0.08(0.05-0.13)  0.22(0.01-0.55)  0.25(0.17-0.32)  0.26(0.09-0.42)  0.08(0.05-0.13)  0.30(0.19-0.43)  0.27(0.19-0.35)  0.06(0.04-0.09)  0.27(0.18-0.37)  0.19(0.10-0.29)  0.05(0.03-0.08)  0.38(0.24-0.54)  0.27(0.15-0.39)  0.33(0.20-0.48)  0.11(0.07-0.16)  0.30(0.19-0.43) | 94.9%  96.4%  84.6%  /  93.2%  91.4%  95.5%  /  /  91.7%  0.0%  95.8%  96.4%  /  /  94.4%  /  49.6%  / | <0.0001  <0.0001  <0.0001  /  0.0001  <0.0001  <0.0001  /  /  <0.0001  0.441  <0.0001  <0.0001  /  /  <0.0001  /  0.158  / |

Abbreviations: AWGS, Asian Working Group for Sarcopenia; EWGSOP, European Working Group on Sarcopenia in Older People; FNIH, Foundation for the National Institutes of Health; SDS, Self-rating Depression Scale; GDS, Geriatric Depression Scale; CES-D, Center for Epidemiologic Studies Depression Scale; B-CIS-R, Brazilian version of the Clinical Interview Scheduled Revised; DSM, Diagnostic and Statistical Manual of Mental Disorders.

Table S4b The results of subgroup analysis for ORs between depression and sarcopenia

| Outcomes | No. of studies | Meta-analysis results | *I*^2^ | *P* |
| --- | --- | --- | --- | --- |
| Diagnostic criteria for sarcopenia  AWGS (2014)  AWGS (2019)  EWGSOP (2010)  FNIH  Measurement method for depression  SDS  GDS-15  CES-D-10  B-CIS-R  DSM  HADS  PHQ9  Body Mass Index  Normal  Overweight  Unknown  Country  China  Korean  Newland  Japanese  Turkey  Brazil  Spain  USA  Thailand | 4  7  7  2  2  10  3  1  2  1  1  7  6  7  5  2  1  5  1  1  1  2  2 | 1.50(1.05-2.13)  1.25(1.10-1.44)  1.55(0.99-2.45)  2.67(1.74-4.09)  1.05(0.99-1.11)  1.25(1.00-1.52)  1.32(1.16-1.52)  2.23(1.11-4.48)  6.09(2.35-9.84)  1.10(1.02-1.19)  2.97(1.11-5.97)  1.06(1.00-1.12)  1.01(0.82-1.21)  1.58(1.07-2.09)  1.25(0.92-1.09)  1.12(0.85-1.39)  0.80(0.60-1.10)  1.06(1.00-1.12)  2.55(1.11-1.18)  2.23(1.11-4.48)  1.10(1.02-1.19)  0.95(0.54-1.36)  2.25(0.81-3.68) | 70.2%  56.4%  90.2%  1.0%  4.0%  83.2%  32.1%  /  39.8%  /  /  24.7%  51.0%  93.4%  48.2%  14.0%  /  29.7%  /  /  /  82.9%  10.0% | 0.009  0.024  <0.0001  0.524  0.701  <0.0001  0.229  /  0.179  /  /  0.232  0.056  <0.0001  0.085  0.281  /  0.201  /  /  /  0.015  0.571 |

Abbreviations: AWGS, Asian Working Group for Sarcopenia; EWGSOP, European Working Group on Sarcopenia in Older People; FNIH, Foundation for the National Institutes of Health; SDS, Self-rating Depression Scale; GDS, Geriatric Depression Scale; CES-D, Center for Epidemiologic Studies Depression Scale; B-CIS-R, Brazilian version of the Clinical Interview Scheduled Revised. BDI-II, Beck Depression Inventory II; HADS, Hospital Anxiety and Depression Scale; PHQ9, Patient Health Questionnaire-9.

Table S4c The results of subgroup analysis in prevalence of depression in possible sarcopenia

| Outcomes | No. of studies | Meta-analysis results | *I*^2^ | *P* |
| --- | --- | --- | --- | --- |
| Diagnostic criteria for sarcopenia  AWGS (2014)  AWGS (2019)  EWGSOP (2010)  Measurement method for depression  SDS  GDS-15  CES-D-10  BDI  PHQ-9  Body Mass Index  Normal  Overweight  Unknown  Country  China  Korean  Japanese  Brazil | 2  3  2    1  2  1  1  2  2  1  4  3  2  1  1 | 0.15(0.07-0.22)  0.14(0.05-0.24)  0.16(0.14-0.18)  0.27(0.17-0.37)  0.14(0.09-0.19)  0.25(0.17-0.32)  0.11(0.09-0.13)  0.12(0.04-0.19)  0.15(0.07-0.22)  0.12(0.08-0.15)  0.16(0.09-0.22)  0.17(0.10-0.24)  0.10(0.08-0.13)  0.27(0.17-0.37)  0.17(0.13-0.20) | 83.9%  92.5%  2.0%  /  64.1%  /  35.3%  93.5%  83.9%  /  92.5%  82.7%  66.8%  /  / | <0.001  <0.001  0.648  /  0.095  /  0.213  <0.001  <0.001  /  <0.001  0.031  0.049  /  / |

Abbreviations: AWGS, Asian Working Group for Sarcopenia; EWGSOP, European Working Group on Sarcopenia in Older People; SDS, Self-rating Depression Scale; GDS, Geriatric Depression Scale; CES-D, Center for Epidemiologic Studies Depression Scale; BDI-II, Beck Depression Inventory II; HADS, Hospital Anxiety and Depression Scale; PHQ9, Patient Health Questionnaire-9.

Table S4d The results of subgroup analysis for ORs between depression and possible sarcopenia

| Outcomes | No. of studies | Meta-analysis results | *I*^2^ | *P* |
| --- | --- | --- | --- | --- |
| Diagnostic criteria for sarcopenia  AWGS (2014)  AWGS (2019)  EWGSOP (2010)  Measurement method for depression  SDS  GDS-15  CES-D-10  PHQ9  Body Mass Index  Normal  Overweight  Unknown  Country  China  Korean  Japanese  Turkey | 2  3  2  2  2  1  2  3  1  3  3  2  1  1 | 1.40(0.75-2.60)  1.75(1.43-2.13)  1.10(0.82-1.48)  1.30(0.80-2.11)  1.12(0.84-1.49)  1.63(0.66-4.06)  1.88(1.34-2.65)  1.44(0.94-2.22)  1.32(0.53-3.31)  1.44(0.91-2.29)  1.65(1.34-2.04)  1.74(0.97-3.14)  1.03(1.00-1.06)  0.99(0.71-1.39) | 76.3%  2.0%  40.0%  93.4%  47.3%  /  40.0%  85.5%  48.0%  73.3%  1.0%  65.9%  /  / | 0.005  0.422  0.196  <0.001  0.149  /  0.456  <0.001  0.165  0.023  0.561  0.032  /  / |

Abbreviations: AWGS, Asian Working Group for Sarcopenia; EWGSOP, European Working Group on Sarcopenia in Older People; SDS, Self-rating Depression Scale; GDS, Geriatric Depression Scale; BDI-II, Beck Depression Inventory II; PHQ9, Patient Health Questionnaire-9.
