# Supplementary material for: The bidirectional association between depression and sarcopenia: a systematic review and meta-analysis
Source: Front Public Health. 2025 Nov 13;13:1673755. doi: 10.3389/fpubh.2025.1673755 (PMC12658358; doi:10.3389/fpubh.2025.1673755)
Supplement: Supplementary file 5 [file Table_5.docx]

Table S5a Characteristics of studies included in the meta-analysis for prevalence of sarcopenia in depression

| First author | Country | Study design | Age  (mean) | No.of depression | No.of  sarcopenia | Prevalence | BMI (mean) | Sarcopenia diagnosis | Depression diagnosis |
| --- | --- | --- | --- | --- | --- | --- | --- | --- | --- |
| Yazar 2019  Yazar 2019  Wang 2018  Delibas 2021 | Turkey  Turkey  China  Turkey | Cross-section  Cross-section  Cross-section  Cross-section | 74.1  75.8  69.3  67.7 | 50  66  71  69 | 9  2  11  32 | 0.18  0.03  0.15  0.46 | 24.9  26.1  23.2  Unknown | EWGSOP (2010)  EWGSOP (2010)  AWGS (2014)  EWGSOP (2010) | GDS-15  GDS-15  GDS-15  GDS-15 |

Abbreviations: AWGS, Asian Working Group for Sarcopenia; EWGSOP, European Working Group on Sarcopenia in Older People; GDS, Geriatric Depression Scale.

Table S5b Characteristics of studies included in the meta-analysis for ORs between sarcopenia and depression

| First author | Country | Study design | Age  (mean) | Sample size | BMI (mean) | OR (95%CI) | Adjust OR (95%CI) | Sarcopenia diagnosis | Depression diagnosis |
| --- | --- | --- | --- | --- | --- | --- | --- | --- | --- |
| Wang 2018  Delibas 2021  Lee 2018 | China  Turkey  Korean | Cross-section  Cross-section  Cross-section | 69.3  68.7  74.3 | 865  204  201 | 23.2  Unknown  24.9 | 2.73(1.35,5.51)  /  4.69(1.13,19.5) | 2.45(1.12,5.45)  0.98(0.90,1.07)  5.49(1.06, 27.9) | AWGS (2014)  EWGSOP (2010)  AWGS (2014) | GDS-15  GDS-15  CES-D-10 |

Abbreviations: AWGS, Asian Working Group for Sarcopenia; EWGSOP, European Working Group on Sarcopenia in Older People; GDS, Geriatric Depression Scale; CES-D, Center for Epidemiologic Studies Depression Scale.
