# Supplementary figures and images for: The bidirectional association between depression and sarcopenia: a systematic review and meta-analysis
Source: Front Public Health. 2025 Nov 13;13:1673755. doi: 10.3389/fpubh.2025.1673755 (PMC12658358; doi:10.3389/fpubh.2025.1673755)

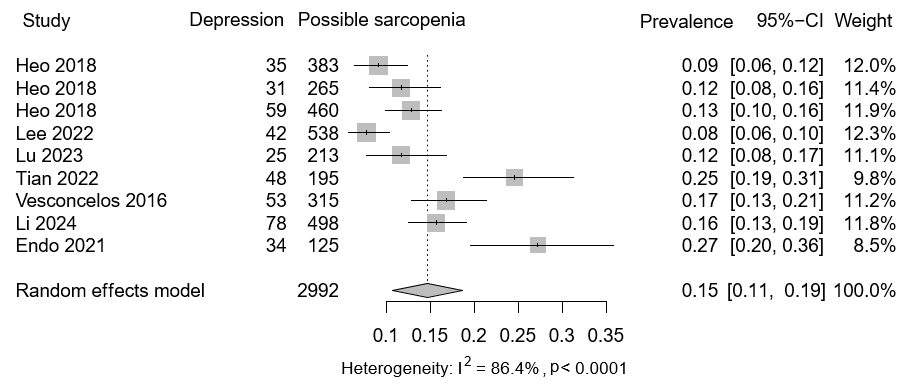

Supplement: Supplementary file 6 [file Image_1.tif]

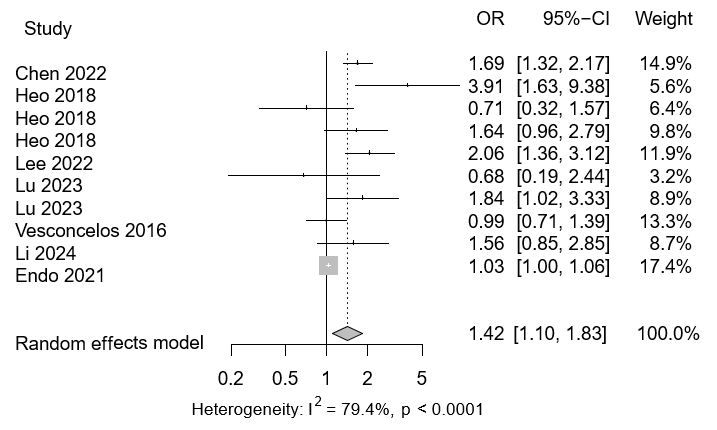

Supplement: Supplementary file 7 [file Image_2.tif]

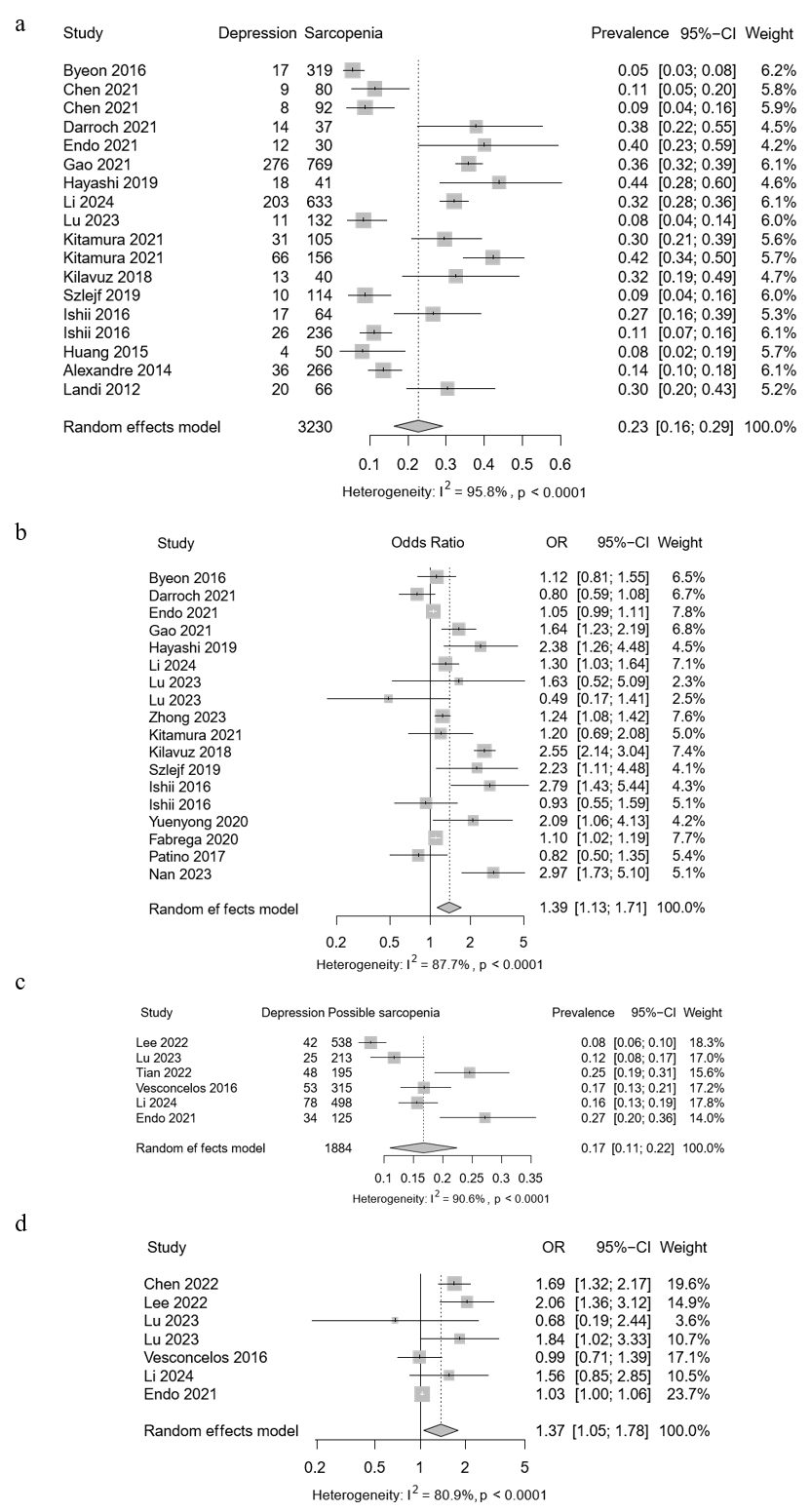

Supplement: Supplementary file 8 [file Image_3.tif]

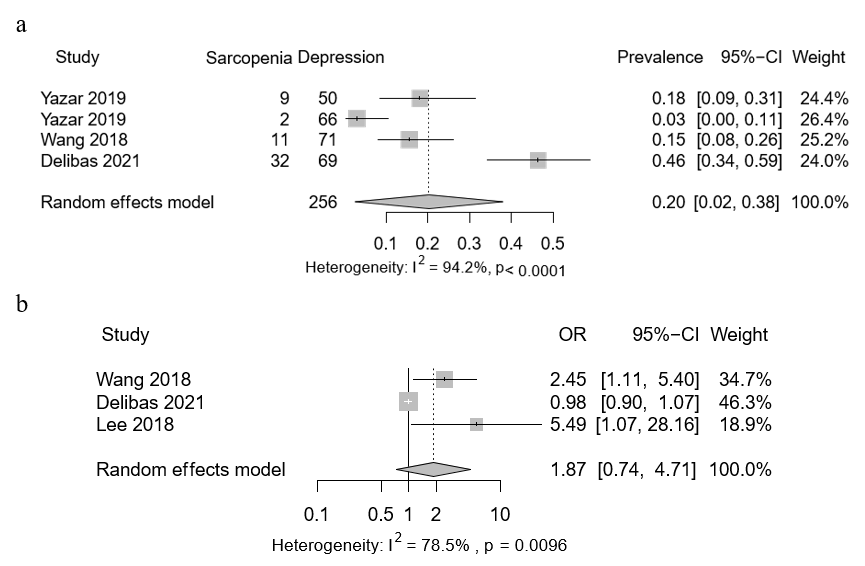

Supplement: Supplementary file 9 [file Image_4.tif]
